# Transjugular Intrahepatic Portosystemic Shunt (TIPS) for Treatment of Bleeding from Cardiofundal and Ectopic Varices in Cirrhosis

**DOI:** 10.3390/jcm13195681

**Published:** 2024-09-24

**Authors:** Sarah Shalaby, Oana Nicoară-Farcău, Valeria Perez-Campuzano, Pol Olivas, Sonia Torres, Juan Carlos García-Pagán, Virginia Hernández-Gea

**Affiliations:** 1 Barcelona Hepatic Hemodynamic Laboratory, Liver Unit, Hospital Clínic, Institut de Investigacions Biomèdiques August Pi i Sunyer (IDIBAPS), 08036 Barcelona, Spain; oana.farcau@gmail.com (O.N.-F.); vperez2@clinic.cat (V.P.-C.); olivas@clinic.cat (P.O.); storresr@clinic.cat (S.T.); jcgarcia@clinic.cat (J.C.G.-P.); 2 Fundació de Recerca Clínic Barcelona (FRCB-IDIABPS), CIBEREHD (Centro de Investigación Biomédica en Red Enfermedades Hepáticas y Digestivas), Health Care Provider of the European Reference Network on Rare Liver Disorders (ERN-RareLiver), 08036 Barcelona, Spain; 3 Hepatology Department, Regional Institute of Gastroenterology and Hepatology “Octavian Fodor”, “Iuliu Hatieganu” University of Medicine and Pharmacy, 3rd Medical Clinic, 400394 Cluj-Napoca, Romania; 4 Departament de Medicina i Ciències de la Salut, Universitat de Barcelona, 08036 Barcelona, Spain

**Keywords:** gastric varices, portal-hypertensive hemorrhage, portosystemic shunt, variceal embolization, secondary prophylaxis

## Abstract

Acute variceal bleeding in cirrhosis represents a critical clinical event that significantly impacts patient prognosis, with mortality rates increasing further after a second episode. This underscores the need for immediate intervention and optimal prophylaxis. The creation of a transjugular intrahepatic portosystemic shunt (TIPS) has been proven to be highly effective for managing esophageal variceal bleeding. However, the use of TIPS for managing cardiofundal gastric varices and ectopic varices remains debated due to their unique vascular anatomy and the limited data available. These varices, although less prevalent than esophageal varices, are complex and heterogeneous vascular shunts between the splanchnic venous system and the systemic veins. Indeed, while endoscopic therapy with tissue adhesives is widely endorsed for achieving hemostasis in active hemorrhage, there is no consensus regarding the optimal approach for secondary prophylaxis. Recent research emphasizes the efficacy of endovascular techniques over endoscopic treatments, such as TIPS and endovascular variceal embolization techniques. This review examines the use of TIPS in managing acute variceal bleeding in patients with cirrhosis, focusing specifically on cardiofundal gastric varices and ectopic varices, discussing optimal patient care based on the latest evidence, aiming to improve outcomes for this challenging subset of patients.

## 1. Introduction

**Figure 1:**
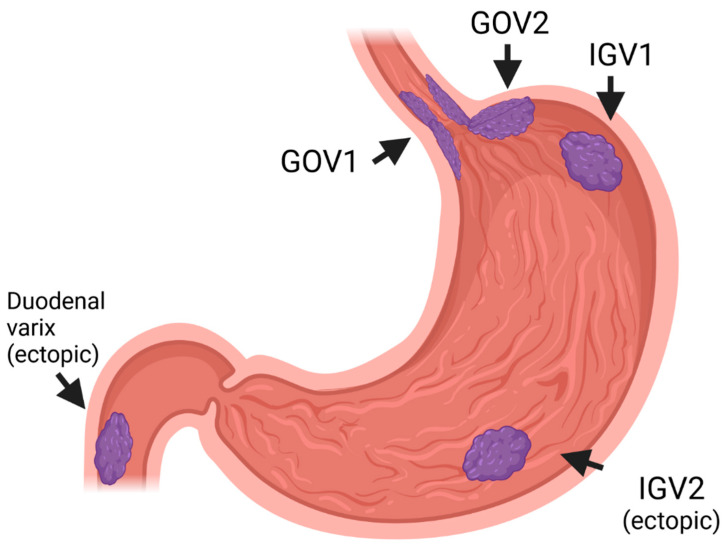
Endoscopic classification of gastric varices according to Sarin Classification. Gastroesophageal varices type 1 (GOV1), gastroesophageal varices type 2 (GOV2), isolated gastric varices type 1 (IGV1), isolated gastric varices type 2 (IGV2), and duodenal varices. Created with Biorender.com.

Acute variceal bleeding (AVB) in cirrhosis represents a critical clinical event significantly impacting patient prognosis, with mortality rates ranging between 10% and 15% and increasing further after a second episode. This underscores the need for immediate intervention and optimal prophylaxis [1,2]. The occurrence of AVB in cirrhotic patients is influenced by several factors such as the severity of portal hypertension (PH), liver function, variceal anatomy, and hemodynamics, emphasizing the importance of tailored patient management, which often requires a multidisciplinary team including hepatogastroenterologists, interventional radiologists, and intensive care specialists. In this setting, the creation of a transjugular intrahepatic portosystemic shunt (TIPS) has been proven to be highly effective for the management of esophageal variceal bleeding, with its application and timing being progressively adapted to suit individual patient characteristics and relevant outcomes [1,3,4,5,6]. However, its utilization for managing cardiofundal gastric varices (GOV2 and IGV1) and ectopic varices remains debated due to their unique vascular anatomy and the limited data available. Indeed, these varices are relatively rare and represent a complex and heterogeneous set of vascular shunts between the splanchnic venous system and the systemic veins. Despite being less prevalent than esophageal varices, bleedings from cardiofundal GV and ectopic varices (Figure 1) tend to be more severe, with higher failure rates of standard prophylaxis and higher associated mortality [7,8,9,10,11]. In this review, we will examine data on the use of TIPS in managing AVB in patients with cirrhosis, focusing specifically on cardiofundal GV and ectopic varices, exploring various strategies to optimize patient care based on the latest evidence.

## 2. Primary Prophylaxis

Non-selective beta-blockers (NSBBs) are the primary choice for preventing bleeding from esophageal varices and carvedilol is currently the NSBB of choice [12,13]. However, there are limited data on primary prophylaxis for cardiofundal GV and ectopic varices, which also have lower bleeding rates compared with esophageal varices [11,14]. A randomized study by Mishra et al. on patients with cirrhosis and cardiofundal GV compared cyanoacrylate injection, propranolol, and no treatment, finding lower bleeding rates with cyanoacrylate but no significant mortality difference between cyanoacrylate and propranolol [14]. Furthermore, Choe et al. conducted a retrospective study in Korea comparing cyanoacrylate injection, balloon-occluded retrograde transvenous obliteration (BRTO), and no treatment [15]. Specifically, 247 patients with cirrhosis and gastric varices were included, 123 of which were cardiofundal GV (36 treated with endoscopic cyanoacrylate injection, 25 with BRTO, and 62 received no treatment). The bleeding rate was highest in the no treatment group, with BRTO showing the lowest bleeding rate and higher eradication rates. Despite these findings, no method significantly reduced mortality from preventing the first bleed. Thus, the study highlights the lack of benefit of primary prophylaxis with these techniques for cardiofundal varices.

Due to the potential higher risks of adverse events of invasive procedures (endoscopic and intravascular), primary prophylaxis with NSBBs is currently recommended for patients with this type of varices despite the scarce available evidence supporting their efficacy. However, the PREDESCI trial concluded that in compensated cirrhosis, NSBBs prevent not only variceal bleeding but also other types of decompensation, including ascites, and increase survival [15]. Even though patients with gastric varices were not adequately represented in this study, portal hypertension remains the common driver for decompensation, regardless of the varices’ location. Furthermore, the latest Baveno consensus has shifted the paradigm from preventing bleeding in patients with varices to preventing decompensation in patients with portal hypertension. The recommendation now is to administer NSBB to all patients with clinically significant portal hypertension, even if varices are absent. Lastly, the potential role of TIPS for the primary prevention of GV and ectopic varices hemorrhage has not been assessed and is not currently recommended.

## 3. General Measures for Acute Portal-Hypertensive Bleeding in Cirrhosis

Similarly, to esophageal varices, general measures for AVB include obtaining adequate intravenous access and airway control, with early endotracheal intubation for patients at risk [16,17]. Vasoactive drugs and broad-spectrum antibiotics, adjusted to reflect local resistance patterns, should be started as early as possible and maintained for 2–5 days after confirmation of PH-related bleeding by urgent therapeutic endoscopy [18,19]. The general correction of coagulation parameters is not recommended [20] as it is well known that they are not the cause of the bleeding and their correction does not have an impact in outcome. Volume expansion with crystalloids and transfusions should be carefully balanced to maintain adequate renal perfusion while avoiding volume overload, as this can further increase portal pressure. The use of colloids should be avoided. Moreover, transfusion or red blood cells should be conducted in a restrictive fashion, as this improves survival (target hemoglobin level of 7 g/dL in hemodynamically stable patients without relevant comorbidities) [1,3,21,22,23]. Endoscopic procedures are essential to identify the source of bleeding and initiate targeted therapy, as well as to stratify the patient’s risk, and it should be performed as soon as safely possible. If the source of bleeding is due to portal hypertension, there is no evidence to support starting or maintaining proton pump inhibitors. They should be discontinued due to their potential increased risk of hepatic encephalopathy and infection [24,25].

## 4. Endoscopic Evaluation and Achievement of Hemostasis

In patients suspected of variceal hemorrhage, current international guidelines recommend endoscopic evaluation within 12 h from the time of patient presentation, provided the patient has been hemodynamically resuscitated [4,26].

### 4.1. GOV1

Endoscopic band ligation (EBL) is recommended as the first choice of treatment to achieve hemostasis in cases of bleeding from esophageal varices and gastroesophageal varices type 1 (GOV1—Figure 1) [1,3,4,21]. Indeed, GOV1 varices are anatomically similar to esophageal varices, as they are an extension of these along the lesser curvature of the stomach. They also have a similar response to treatment. In the case of massive hemorrhage, balloon tamponade with a Sengstaken–Blakemore tube or a self-expanding fully coated metal esophageal stent (SEMS) [4,27] may be used as a bridge to other treatments or before referring the patient to experienced centers.

### 4.2. Cardiofundal Varices (GOV2 and IGV1)

In cases where the bleeding source is a cardiofundal GV (GOV2—esophageal varices extending down to gastric fundus—or IGV1—located in the fundus of the stomach without connection with the esophagus—Figure 1), endoscopic treatment options include cyanoacrylate glue injection, sclerotherapy, EBL, and thrombin injection. Tissue adhesives have been shown to be more effective than EBL and sclerotherapy in controlling acute bleeding [28,29] and there is a widespread agreement supporting the use of cyanoacrylate injection to achieve hemostasis in the acute setting (acute bleeding control rates of 87–93%) [1,3,4,7,21,30,31,32,33].

The most reported and feared complication of cyanoacrylate injection is glue embolization leading to pulmonary embolus [34] and other complications include ulceration at the injection site of the varix, the extrusion of the glue cast, and sepsis, with a complication-related mortality of 0.5% [30,34]. Recently, the combination of endoscopic ultrasound-guided coil embolization with cyanoacrylate glue injection has been proven to outperform monotherapy, showing greater technical success and fewer complications [35,36,37], and has been endorsed by the European Society of Gastrointestinal Endoscopy for managing bleeding GV in experienced centers [4]. Nevertheless, the choice of treatment method depends on the specific clinical scenario, local expertise, and patient conditions. Although tissue adhesive injection may eradicate cardiofundal varices, about one-third of patients experience rebleeding, necessitating rescue treatments [38].

In severe bleeding cases and uncontrolled bleeding, balloon tamponade [4,27] may be required to stop bleeding and act as a bridge to further treatments and/or facilitate referral to specialized centers. A Linton–Nachlas tube is specifically designed for GV and it is more effective than the Sengstaken–Blakemore tube in achieving hemostasis due to the large volume of its single gastric balloon (600 mL), which allows adequate compression of the gastric fundus [39]. However, if the Linton–Nachlas balloon is not available, compression with the gastric balloon of a maximally inflated Sengstaken–Blakemore tube may be an option. SEMS are not useful in this context due to the lack of fundal coverage.

### 4.3. Ectopic Varices

Ectopic varices can appear in any part of the gastrointestinal tract other than the esophagus or the gastric fundus (Figure 1). They are most commonly found in the duodenal–jejunal region and the colorectum, but they may also appear at stoma sites, in the small intestine, and very rarely, in the urinary bladder wall, gallbladder, and biliary duct [9]. Abdominal and pelvic surgeries, and the creation of enterostomies, are significant risk factors because postoperative adhesions can obstruct blood flow and facilitate the development of portosystemic collaterals [40]. Although uncommon, ectopic varices are prevalent in patients with portal hypertension [9,41] and account for 1% to 5% of all variceal bleeding cases with high associated mortality [42,43]. Diagnosing and treating ectopic varices presents significant challenges due to the difficulty or impossibility of endoscopic visualization, which limits the feasibility of standard endoscopic treatment options. Even when accessible via endoscopy, treatments such as EBL, sclerotherapy, cyanoacrylate glue injection, thrombin injection, and embolization with glue and coils show varying success rates and carry a high risk of recurrence, rebleeding, and ulceration [9,44,45,46]. Consequently, it has been proposed that endoscopic therapy might serve best as a temporary measure until more definitive treatment becomes available. Radiological techniques have been suggested as potentially effective for achieving hemostasis and secondary prophylaxis [47,48], although further studies are needed to determine the optimal treatment approach.

## 5. Achievement of Hemostasis and Secondary Prophylaxis

The probability of experiencing rebleeding after a first AVB episode within the first year can reach 60% if left untreated, with up to 50% mortality rates [49]. Thus, once initial bleeding control is established, it becomes crucial to optimize secondary prophylaxis, tailoring it to the varices’ type, their anatomical characteristics, patient-specific factors, and the expertise of the treating center.

### 5.1. GOV1

**Figure 2:**
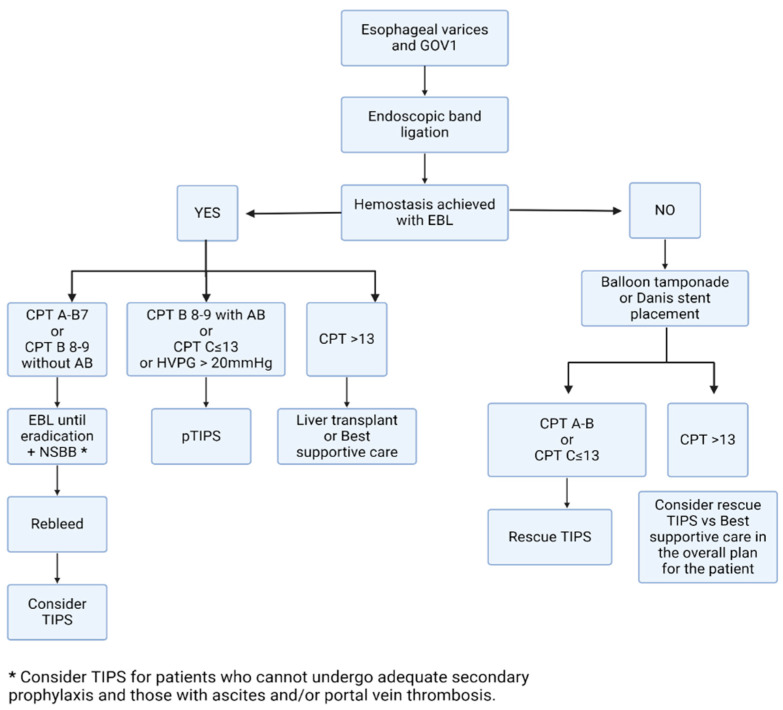
Algorithm summarizing current recommendations for secondary prophylaxis for esophageal varices and GOV1. Created with Biorender.com.

The combination of NSBB and EBL up to variceal eradication is the gold standard therapy for secondary prophylaxis of GOV1, as for esophageal varices, which has proven to be more effective than either therapy alone [50]. GOV1 can be treated similarly to esophageal varices as they share venous drainage through the left gastric vein into the portal vein and are connected to the azygos system. In this context, the creation of a TIPS directly affects the pressure gradient in this very anatomical site and has been historically established as the treatment of choice in uncontrolled bleeding following the failure of secondary prophylaxis (salvage/rescue TIPS) [1,3,4,21]. Nevertheless, the preemptive use of TIPS (pTIPS), placed during the bleeding episode but once hemostasis is achieved, is now recommended by all major international guidelines due to its high efficacy in preventing early rebleeding and reducing mortality in patients who survive the initial AVB episode. The challenge lies in accurately identifying patients at a high risk of early rebleeding, carefully balancing pTIPS benefits against the potential risks of the procedure. Indeed, the definition of “high-risk” for early rebleeding is constantly evolving [51], and currently incorporates a combination of simple clinical and endoscopic criteria: a Child–Turcotte–Pugh (CTP) score of B = 8–9 with active bleeding at the initial endoscopy despite the use of vasoactive drugs, or a CTP score of C ≤ 13 [52,53,54,55,56], and hemodynamic criteria: hepatic venous pressure gradient > 20 mmHg [57]. The rationale behind pTIPS is to prevent failure and early rebleeding, both associated with a detrimental impact in prognosis. As the risk of rebleeding concentrates in the first days after the indicial bleeding, TIPS should be performed as soon as possible after achieving hemodynamic stability and preferable in centers of expertise [55]. For patients with relatively preserved liver function (CTP A, CTP B7) or CTP B 8–9 without active bleeding, while pTIPS can reduce rebleeding risk, the impact on survival has not been demonstrated and its indication should be considered on a case-by-case basis. A recent large multicenter study proposed a nomogram to identify high-risk patients also within this group by combining baseline CTP score, creatinine, and sodium, which might be validated in future studies [58]. Finally, TIPS is currently recommended only as a life-saving measure for patients with CTP C > 13, due to the high risk of acute-on-chronic liver failure and high 6-week mortality rates [59,60,61,62,63,64]. Thus, its use should be considered as part of the overall therapeutic plan for these patients, especially in the context of liver transplant [63,65,66]. Figure 2 summarizes the current algorithm for secondary prophylaxis for GOV1, which is the same as esophageal varices.

### 5.2. Cardiofundal Varices (GOV2 and IGV1)

As per esophageal and GOV1 varices, patients who survive an initial episode of AVB from cardiofundal GV (GOV2/IGV1) face a high rebleeding risk (up to 65%) despite receiving initial endoscopic treatment, with a high risk of mortality. This significant risk underscores the necessity for continued variceal management and robust secondary prophylaxis [7,11]. While advancements have been made in repeated endoscopic injection treatments, which are the most frequently applied secondary prophylaxis method combined with NSBB [33], recent decades have brought attention to radiological interventional techniques, such as TIPS and direct variceal embolization, as potentially more effective long-term alternatives. Even though the rationale to use these techniques is solid, the superiority of these methods over endoscopic ones has not been definitively proven through proper randomized controlled trials that consider only cardiofundal GV, nor fully evaluated against their different side effects [67].

#### 5.2.1. Evidence Supporting the Use of TIPS

**Figure 3:**
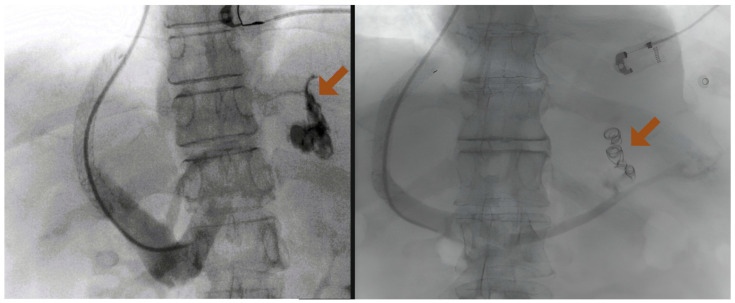
**Left panel:** Example of TIPS placement for bleeding from cardiofundal GV, showing previous treatment with cyanoacrylate (arrow). **Right panel:** Example of TIPS placement for bleeding from cardiofundal GV with concomitant antegrade embolization of the varix (arrow). Created with Biorender.com.

A retrospective study from the UK has shown that the rate of rebleeding within 30 days is lower for patients treated with TIPS compared with cyanoacrylate [68]. However, this study combined cardiofundal GV with GOV1, and no significant differences in survival, morbidity, or complications were observed. Below, we present evidence supporting the use of TIPS in various scenarios, with specific emphasis on cardiofundal gastric varices in patients with cirrhosis (Figure 3). Additionally, studies exclusively dedicated to cardiofundal gastric varices are summarized in Table 1.

##### Preemptive TIPS

Previously discussed studies on the use of pTIPS included only a small number of patients with GOV2 and none of the reported patients had IGV1, making it difficult to extrapolate evidence for this group of varices. To date, only one small randomized controlled trial has been published for cardiofundal GV. In their study, Escorsell et al. found that implementing pTIPS within 1 to 5 days following hospital admission could significantly improve rebleeding-free survival rates in patients with CTP B-C cirrhosis experiencing cardiofundal GV bleeding [69]. However, due to the rarity of these varices and the impact of the COVID-19 pandemic, the study was unable to enroll a sufficient number of participants to adequately power the investigation for the selected outcomes, thereby compromising its conclusions. France is currently conducting another multicenter trial (NCT03705078) aiming to provide additional evidence on the use of pTIPS in this context.

##### Salvage/Rescue TIPS

The evidence for the use of rescue TIPS providing specific data for cardiofundal GV dates from 1998, using bare stents [70]. In a more recent study by Jalan et al., which included a substantial number of cardiofundal GV, it was confirmed that the efficacy of rescue TIPS in this setting is comparable to that observed for esophageal varices [75]. Although TIPS is highly effective in achieving hemostasis in patients with uncontrolled bleeding from cardiofundal GV, reported mortality rates remain high. A recent multicenter study evaluated patients treated with salvage TIPS for refractory variceal bleeding, including more than 20% of patients with gastric varices, although the subtype was not reported. The study identified lactate levels ≥12 mmol/L and/or a MELD score ≥ 30 as factors associated with mortality >90%, which are now recommended as futility criteria in the setting of variceal bleeding [66].

##### Elective TIPS for Secondary Prophylaxis

In a single randomized controlled trial comparing TIPS with cyanoacrylate glue injection, it was found that patients receiving TIPS with bare stents, following initial bleeding control with cyanoacrylate, had lower rebleeding rates from cardiofundal GV, while survival and hepatic encephalopathy rates remained comparable [73]. Other observational studies have also shown high efficacy rates for TIPS use [68,76]; however, these ones did not distinguish between outcomes for cardiofundal GV and GOV1. Indeed, reported rebleeding rates in studies considering exclusively cardiofundal GV (Table 1) remain above 10% despite TIPS patency and combination with variceal embolization (performed when persistent filling of the cardiofundal GV was observed at post-TIPS venography) [71,72], underlying the need to perform dedicated studies in this context. Thus, even though recent guidelines advocate for TIPS as a first-line treatment to prevent rebleeding of cardiofundal GV based on this evidence [1,3,4,21], there is need for further data to reinforce recommendations and adequately select the best treatment for each patient.

##### Antegrade and Retrograde Variceal Embolization vs. TIPS

**Figure 4:**
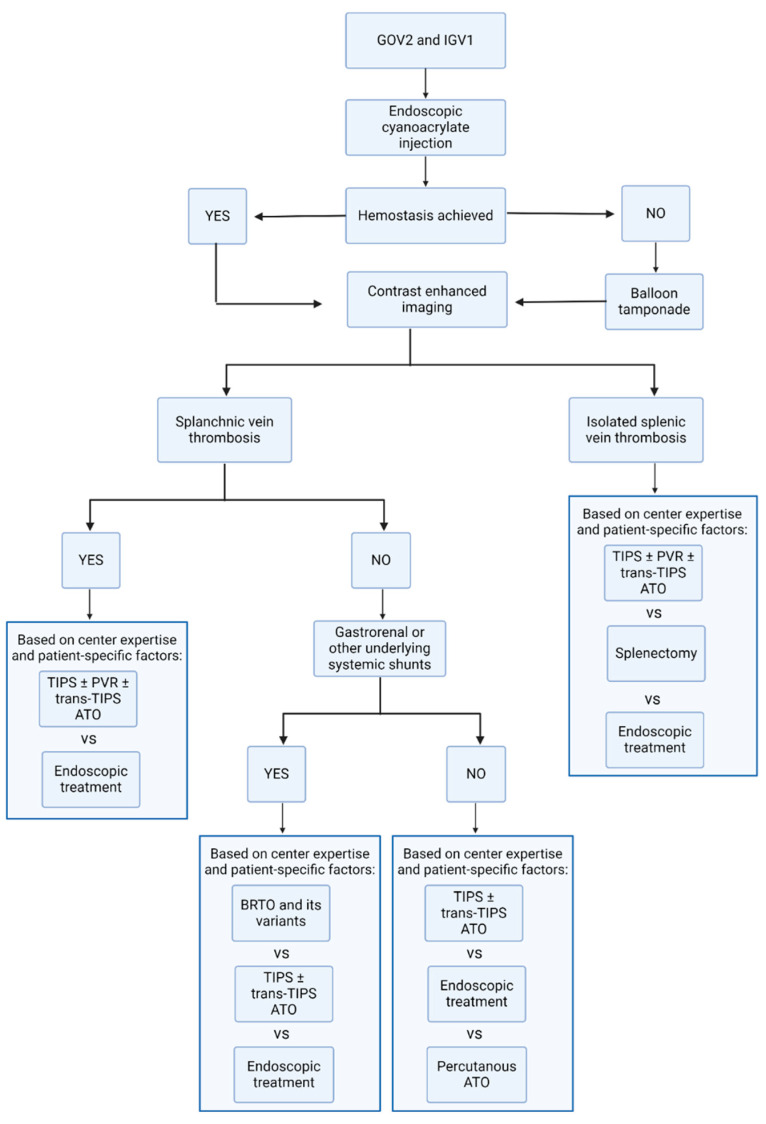
Algorithm summarizing current recommendations for secondary prophylaxis for cardiofundal GV. Created with Biorender.com.

The majority of cardiofundal GV receive blood from the left gastric, posterior, and short gastric veins, and a gastro-renal shunt (GRS) serves as the primary outflow tract in 80–85% of cases [77], creating a unique hemodynamic profile. This profile is usually characterized by a low-pressure gradient between the splenic and renal veins due to the GRS’s large diameter, which facilitates high blood flow despite relatively low portal pressure gradients [75,78,79,80,81,82], and this could explain rebleeding occurring despite the correct functioning of the TIPS. In this context, direct variceal obliteration techniques seem to outperform TIPS in terms of efficacy, possibly because it directly targets the underlying shunt [83]. These techniques have been employed mainly in the Eastern countries to manage variceal bleeding by injecting a sclerosing agent directly into the varices, causing thrombosis and the eventual obliteration of the variceal lumen. They can be distinguished into antegrade techniques (ATO), which involve advancing a catheter through the portal vein towards the varices and accessing the varix from its afferences, and retrograde obliteration (RTO), which accesses the varices from the systemic venous system, facilitating the injection of the sclerosant in a retrograde manner in those varices that present with a systemic outflow. Balloon-occluded retrograde transvenous obliteration (BRTO) is a specific retrograde technique that enhances sclerosant retention by temporarily occluding the outflow tract with a balloon. BRTO offers other advantages over TIPS, such as reduced hepatic encephalopathy and the potential prevention of liver function deterioration related to portal systemic shunting. Moreover, its variations, such as CARTO (Coil-Assisted Retrograde Transvenous Obliteration) and PARTO (Plug-Assisted Retrograde Transvenous Obliteration), are also effective alternatives, though currently underutilized [84,85,86,87,88]. RTO techniques are particularly favored in Eastern countries and specialized centers, but their complexity and logistical challenges hinder their broader adoption. Moreover, both RTO and ATO may lead to an increase in portal pressure (due to the closure of a large portosystemic shunt underlying the varix), potentially exacerbating PH-related complications like esophageal varices and ascites. As a result, current guidelines recommend a cautious approach to RTO and advise for further assessments of its role in managing GVs [1,3]. Finally, RTO is not feasible without a GRS (or similar shunts), and both RTO and ATO are contraindicated in cases of splanchnic vein thrombosis, whereas TIPS remains a viable alternative. Thus, TIPS is generally preferred in the absence of GRS, among patients with concomitant high-risk esophageal varices, those with previous variceal bleeding episodes, and those suffering from difficult-to-control ascites. Conversely, variceal embolization techniques might be preferred in the presence of GRS, overt hepatic encephalopathy, or when TIPS is contraindicated. Following this rationale, the choice between RTO and TIPS should therefore be based on the anatomy of the variceal inflow and outflow, liver function, and the presence of other complications of portal hypertension. However, the final decision still mainly relies on local expertise, which often does not include all these options, and this might also explain the lack of trials or proper comparative studies proving the value of this theoretical algorithm (Figure 4). Although current guidelines recognize the rationale for using interventional radiology in this context, they leave treatment choices to local expertise due to insufficient data. Further details on the efficacy and safety of ATO/RTO alone in the management of cardiofundal GV go beyond the scope of this review and have been reviewed extensively elsewhere [5].

##### TIPS Combined with Antegrade or Retrograde Variceal Embolization

Combining TIPS with variceal obliteration has also been proposed to enhance efficacy rates in rebleeding prevention in cardiofundal GV (Figure 3) [5]. Recent studies focusing on these varices have shown that combining anterograde embolization with TIPS placement may further reduce bleeding risk [71,72]. This suggests that the combination of both treatments may be more beneficial compared with single therapy. Additionally, a recent small randomized controlled trial indicated that concurrent embolization of large spontaneous shunts during TIPS placement reduces the risk of hepatic encephalopathy in patients with variceal hemorrhage (predominantly from cardiofundal GV) [89], although no effect on rebleeding rate was demonstrated. Another recent retrospective study from China [74], conducted on a large cohort of patients treated with TIPS (using covered stents all dilated to 8 mm) for GV bleeding, identified adjunctive embolization as an independent factor influencing rebleeding and post-TIPS hepatic encephalopathy in cardiofundal GV. However, survival rates were not affected, and the study did not report the protocol for concomitant variceal embolization, the causes of rebleeding, or the rate of TIPS dysfunction, leaving the real impact of embolization in preventing cardiofundal GV rebleeding unclear and open to further discussion. Future research should aim to design prospective studies to properly address this question as the rationale of combining liver resistance decompression (TIPS) with variceal obliteration (ATO/RTO) theoretically allows for the benefits of both methods to be utilized, rather than selecting one exclusively [90]. Indeed, the closure of the GRS or similar shunts can theoretically lower the incidence of post-TIPS encephalopathy compared with using TIPS alone, whether the potential rise of portal pressures also caused by the closure of this shunt (which might lead to worsening/appearance of ascites and esophageal varices) can be prevented by simultaneously or sequentially placing a TIPS. Moreover, TIPS patency might also improve as the closure of competitive shunts can enhance intrahepatic inflow [91]. Moreover, also performing TIPS first offers technical advantages, including better visualization of GV anatomy through a trans-TIPS portal venogram, enabling more effective obliteration planning and improved sclerosant trapping, thereby reducing the risk of migration. Nevertheless, despite the multiple suggestions that combining TIPS and ATO/RTO might be more effective in preventing rebleeding and the other complications of portal hypertension, a higher level of expertise is needed and the supporting data are still limited to individual cases, necessitating more evidence to strengthen recommendations.

Lastly, endoscopic cyanoacrylate injection can also be considered following TIPS to consolidate prophylaxis and achieve variceal obliteration; however, after direct shunt of the porto-systemic circulation, this poses increased risks for complications such as glue embolization. Therefore, endovascular techniques are considered a more favorable option for combination treatment [92].

In summary, pre-treatment imaging using the portal venous phase of contrast is crucial to identify the varices’ location, delineate the specific anatomy of afferent and efferent vessels, and assess the splanchnic vessels’ permeability, to determine the best approach [92]. The choice between ATO/RTO and TIPS should then be based on the anatomy of the variceal inflow and outflow, liver function, and the presence of other complications of portal hypertension. More data on the combination of these techniques should be provided but can potentially represent the best option for secondary prophylaxis in cardiofundal GV. Currently, local expertise often dictates the final decision, explaining the lack of trials or proper comparative studies proving the value of this theoretical algorithm. Thus, even though current guidelines recognize the rationale for using interventional radiology in this context, they leave treatment choices to local expertise due to insufficient data.

### 5.3. Ectopic Varices

Due to their rarity and heterogeneity, evidence on the treatment of ectopic varices is limited, and their unique features and anatomical locations further complicate their management. Rebleeding after initial endoscopic hemostasis is common, with recurrence rates reported up to 80% within 6 months [93,94,95], highlighting the need for additional interventions. Moreover, TIPS alone may not be sufficient either. Indeed, ectopic varices, due to their anatomy and distal location to the portal vein, may bleed also at lower pressure gradients than the standard post-TIPS hemodynamic target. To date, there are six relatively large series (summarized in Table 2) [93,95,96,97,98,99] involving 147 patients with cirrhosis bleeding from ectopic varices treated with TIPS: 120 were treated with TIPS alone and 27 had their varices embolized together with TIPS creation. The rates of rebleeding from ectopic varices varied from 11% to 37%, and 16 patients rebled despite functioning TIPS (final PPG < 12 mmHg), which in most cases were resolved after variceal embolization. According to this evidence, international guidelines suggest proceeding directly to the embolization of these varices concurrently with the creation of a TIPS. Despite this approach, some cases of rebleeding have been reported, although less frequently compared with TIPS alone. Oey et al. reported the largest multicentric study including 53 patients with ectopic varices receiving TIPS for bleeding (85% with covered stents) and a 23% rebleeding rate, with one-fourth presenting functional TIPS at rebleeding [93]. Factors associated with rebleeding included high MELD score (HR: 1.081 per point), ectopic varices at sites other than an enterostomy, and local endoscopic treatment preceding TIPS, while variceal embolization did not significantly improve outcomes. Nevertheless, the protocol for concomitant variceal embolization was not reported, and due to the limited number of patients treated, the value of embolization as an adjunctive measure remains unclear.

Available evidence suggests that ectopic varices may require a more aggressive approach than esophageal ones. This involves careful contrast imaging to plan treatment tailored to the variceal and patient characteristics, aiming for complete obliteration rather than partial embolization. Achieving this may necessitate combining anterograde and retrograde techniques, though this can be challenging due to anatomical complexities. Additionally, complete obliteration may be difficult due to multiple venous connections. Therefore, ideal strategies for treating bleeding from ectopic varices are still debated, and endoscopic monitoring should continue after TIPS creation. Evidence on the use of TIPS for bleeding ectopic varices in patients with cirrhosis is discussed further below, with studies providing specific details summarized in Table 2.

#### 5.3.1. Duodenal Varices

Despite their rarity, mortality rates for bleeding from duodenal varices exceeds 40% [9] and post-TIPS rebleeding risk rate reached 50% in the largest case-series by Oey et al. [93]. In this study, the authors found that local endoscopic treatment preceding TIPS was associated with increased risk of rebleeding and hypothesized that multiple unsuccessful endoscopic therapies attempted before the creation of TIPS, including repeated tissue glue injections, might result in significant duodenal ulcerations increasing the risk of rebleeding. However, this hypothesis needs to be proven. International guidelines currently recommend proceeding with TIPS, both with and without embolization, as well as percutaneous ATO or RTO, after the careful evaluation of patient’s liver function and severity of portal hypertension [1,3,4,5,21]. Thus, until further evidence is available, the treatment should be personalized on a case-by-case basis, taking into account the patient’s specific anatomy and clinical characteristics.

#### 5.3.2. Jejunal/Intestinal Varices

Small bowel variceal bleeding typically presents with the triad of portal hypertension, previous abdominal surgery, and melena of an unidentified source [100], and recent reports indicate that ATO and RTO are effective in controlling this type of bleeding [100,101,102]. Evidence on treatment with TIPS ± variceal embolization is scarce and limited to dated studies using bare stents.

#### 5.3.3. Stomal Varices

Stomal varices occur at the mucocutaneous border of the ostomy due to anastomoses between the high-pressure portal venous system and the systemic venous vasculature of the adjacent abdominal wall [103,104]. Like other ectopic varices in the gastrointestinal tract, the chances of bleeding from these varices are low (3–5%), but such episodes can be severe with high related mortality risk [103,105,106]. In the study by Oey, TIPS was particularly effective in patients with stomal varices compared with non-stomal locations (HR: 9.770; 95% CI: 1.241–76.917; *p* = 0.030) [93]. However, evidence from a review of 163 patients with stomal variceal bleeding [107,108] suggests that a combination of TIPS with other treatments may also be considered.

#### 5.3.4. Rectal Varices

Rectal varices in patients with portal hypertension are distinguished from hemorrhoids by their presence above the dentate line and their origin from tributaries of the inferior mesenteric vein and intrinsic rectal venous plexus. Even though bleeding is rare, about 10% experience severe bleeding that is difficult to control [44,109]. There is no universally accepted treatment protocol for bleeding rectal varices. However, endoscopic treatments like band ligation, injection sclerotherapy, and coil embolization, often used in conjunction with endoscopic ultrasound, have shown some success [110,111,112] and might serve as temporary treatment. Surgical techniques such as direct suturing and stapling are generally ineffective for long-term outcomes and are discouraged. Current international guidelines recommend TIPS combined with variceal embolization as the primary treatment for rectal varices; however, reported success rates for bleeding control are quite variable, ranging from 67% to 79% [93,95,97,98]. In recent years, ATO and RTO have emerged as alternative options, particularly for patients with preexisting encephalopathy or compromised liver function [113,114]. The combination of the two techniques should be also evaluated in future studies.

## 6. Conclusions

The management of AVB from cardiofundal GV and ectopic varices in cirrhosis is complex and remains a subject of debate due to limited evidence. While TIPS is a well-established treatment for esophageal variceal bleeding and has also shown clear benefits in preventing rebleeding in this setting, cardiofundal GV and ectopic varices pose additional challenges due to their unique vascular anatomy and higher risk of severe bleeding, even when the target post-TIPS pressure gradient is achieved. Adding embolization to TIPS appears to reduce the risk of rebleeding although more evidence is needed to confirm this benefit. This underscores the necessity for an expert multidisciplinary approach to create tailored treatment plans. Combining different techniques can enhance efficacy and balance side effects but requires a high level of expertise to manage the increased complexity and risk of intraprocedural complications. Further research is essential to optimize and simplify endovascular approaches, ultimately improving outcomes for this high-risk population.

## Figures and Tables

**Table 1:** Studies reporting results on the use of TIPS in fundal GV bleeding in patients with cirrhosis.

	Baseline							TIPS Procedure					Outcomes				
Study	Patients	TIPS Indication	Liver Disease Etiology	MELD and CHILD (%)	Type of Fundal Varix	PVT	Follow-Up	Stent	PPG (Pre–Post) Specify If Review Was Performed	Combination with Other Endovascular Procedure	Technical Success Rate	Stent Dysfunction Rate	TIPS-Related Complications (HE, Cardiac Failure, ecc)	Post-TIPS Rebleeding	Further Treatments for Rebleeding	Mortality	Relevant Details
Escorsell, 2023 Randomized Controlled Trial [69]	11	Pre-emptive	ALD 5 (46%) HCV 1 (9%) HCV+ALD 1 (9%) MASH 2 (18%) Other 2 (18%)	MELD NA CHILD A/BC 4/7 (36/64)	GOV2 = 6 IGV1 = 5	NA	Mean 14 ± 12 months	ePTFE-covered stent. dilated to either 8 or 10 mm in all cases.	Pre = 17 mmHg (range9–20) Post = 8.5 mmHg (range 4.5–13.5)	Variceal embolization: 4/10 (40%) Reasons: - Post-TIPS PPG > 12 mmHg = 1; - Post-TIPS persistent filling of large collaterals = 3	90% (9/10)—1 death before TIPS creation	NA	HE:1 (10%). Hepatic failure: 2 (20%) Portopulmonary syndrome (10%)	0%	-	1-year bleeding-free survival: 90% (intention-to-treat); 100% (per-protocol)	Pre-emptive TIPS showed better outcomes than drug therapy plus glue injection per protocol analysis, but this was not statistically significant in intent-to-treat analysis due to the study’s small sample size.
Chau TN, 1998, retrospective [70]	28	Rescue	ALD 15 (54%) Viral 7 (25%) PBC 3 (11%) Cryptogenic 2 (7%)	MELD NA CHILD A/B/C 1/10/17 (4/36/60)	NA	No	Median 210 days (range, 48–1272)	Bare Stent Wallstent or Memotherm stents Diameter 8, 10, or 12 mm depending on final PPG, age, and previous-HE	Pre = 20 mm Hg (range 9–37) Post = 10 mm Hg (range 4–25)	No	96.4%	25%	Intra-abdominal bleeding:2/28 HE:1 (3%). Hepatic failure: 3 (11%)	Early (<7 d) 3.6% Late (>7 d) 10.7%	TIPS revision for dysfunction	Over all 12 (43%) 7-day, 21%; 30-day, 42%; 6-month 42%	High prevalence of pre-TIPS end-stage liver disease: 54% with moderate-severe ascites 36% with grade III/IV HE 25% needing mechanical ventilation 4% with HRS Causes of death: Intrabdominal bleeding = 2 Hepatic failure = 2 Sepsis = 5 Rebleeding = 1 HRS = 1
Yu, 2019, retrospective [71]	82	Rescue = 3 Secondary prophylaxis = 79	ALD 10 (12%) Virus 49 (60%) Other 23 (28%)	MELD mean 9.3 ± 3.9 CHILD A/B/C 35/35/12 (43/43/14)	GOV2 = 56 IGV1 = 26 Gastrorenal shunt present in 92.7%	17%	Mean 21.9 ± 12.4 months	ePTFE-covered stent. 8-mm = 4, 10-mm = 78	Pre = 21.4 ± 6.5 mmHg Post = 10.2 ± 3.4 mmHg	Variceal embolization 55/82 (67.1%) Reasons: - acute variceal bleeding - operators’ discretion, based on the number and size of varices, and post-TIPS angiographic filling	100%	11%	HE 27 (34%) Other: NA	16% TIPS+E vs. TIPS: 1 and 2-year (4% and 13% vs. 16%, and 28%; p = 0.041).	Medical treatment or cyanoacrylate injection	16 (19.5%) TIPS+E vs. TIPS: 1 and 2-year 94.5% and 82.3% vs. 84.7% and 84.7%	5 patients rebled despite stent patency Causes of death: hepatic failure = 6, Rebleeding = 4, HCC = 2, HE = 2, multiple organ failure = 1, unknown = 1
Sabri, 2013, retrospective [72]	27	Secondary prophylaxis	ALD 8 (30%) HCV 4 (15%) HCV+ALD 5 (19%) Cryptogenic 4 (15%) PBC 1 (3%) Hemochromatosis 1 (3%) MASH 4 (15%)	MELD median 13.1 (6–28) CHILD NA	All IGV1	No	Median 19.5 months (range 1–52)	ePTFE-covered stent, dilated to 8 mm or 10 mm to achieve PPG < 12 mm Hg	Pre = 16 mmHg (range 12–44) Post = 6 mmHg (range 3–12)	Variceal embolization 12/27, (44%) Reason: persistent variceal filling at post-TIPS venography	100%	NA	HE: 6 (22%) Other: NA	11% at 12-months	TIPS revision for dysfunction = 2 BRTO = 1	26% at 12 months	One patient rebled presenting with hemodynamic instability despite post-TIPS PPG = 7 mmHg and previous embolization of short gastric veins → successfully treated with BRTO
Lo, 2007, prospective randomized controlled trial [73]	16	Secondary prophylaxis	NA	NA	14 GOV2 2 IGV1	No	NA	Bare Stent Wallstent dilated to 8 mm to achieve PPG <10 mmHg when possible	NA	No	100%	23%	NA	NA	NA	0% GOV2 NA for IGV1	Rebleeding rate was 0% for GOV2 in TIPS groups compared with 16% (3/19) in patients that were randomized to endoscopic treatment (cyanoacrylate)
Xia, 2024, retrospective [74]	200	GOV2 patients: Rescue = 20 Secondary prophylaxis = 125 IGV1 patients: Rescue = 17 Secondary prophylaxis = 38	Viral 77 (39%) Other (non-specified) 123 (61%)	GOV2 patients: MELD median TIPS+E: 10 (8–11) and TIPS alone: 9 (8–12) CHILD A/B/C 49/88/8 (34/61/5) IGV1 patients: MELD median TIPS+E: 11 (8–14) and TIPS alone: 10 (9–12) CHILD A/B/C 29/18/8 (53/33/14)	145 GOV2 55 IGV1	No	Median 47.6 months	8 mm covered stent, Viatorr TIPS Endoprosthesis (Gore Medical) or Fluency stent (BD)	GOV2 patients: TIPS+E: Pre = 24 mmHg (range 20–27) Post = 9 mmHg (6–12) TIPS alone: Pre = 22 (19–26) Post = 7 mmHg (5–11) IGV1 patients: TIPS+E: Pre = 19 mmHg (15–23) Post = 5 mmHg (4–8) TIPS alone: Pre = 18 mmHg (15–20) Post = 4 (2 = 10)	Variceal embolization in 103 GOV2 (71%) and 42 IGV1 (76%) Reason: at the discretion of the primary operators, based on the number and size of the feeding and draining veins of the GVs	100%	NA	HE = GOV2: TIPS alone 51.5% vs. TIPS+E 31.8%; HR, 0.47; 95% CI, 0.27–0.82; *p* = 0.008; IGV1: 38.5% vs. 11.6%; HR, 0.25; 95% CI, 0.07–0.92; *p* = 0.04)	GOV2 patients: TIPS alone 25.1% vs. TIPS+E 7.8%; HR, 0.26; 95% CI, 0.09–0.74; *p* = 0.01). IGV1 patients: TIPS alone 30.8% vs. TIPS+E 5.6%; HR, 0.15; 95% CI, 0.03–0.84; *p* = 0.03)	NA	GOV2 patients: TIPS alone 9.9% vs. TIPS+E8.8%; HR, 0.77; 95% CI, 0.23–2.57; *p* = 0.68), IGV1 patients: TIPS alone 8.3% vs. TIPS+E13.0%; HR, 1.56; 95% CI, 0.18–13.35; *p* = 0.69)	Patients who underwent adjunctive embolization had higher post-TIPS PPG than those who had TIPS alone in cases of GOV2 and IGV1, but not GOV1 Adjunctive embolization was identified as an independent influencing factor for rebleeding and HE, but not for mortality

Legend: ALD: Alcohol-associated Liver Disease, HCV: Hepatitis C Virus, MASH: Metabolic dysfunction-Associated Steatohepatitis, PPG: Portal Pressure Gradient, HE: Hepatic Encephalopathy, PBC: Primary Biliary Cholangitis, HRS: Hepatorenal Syndrome, HCC: Hepatocellular Carcinoma, MELD: Model for End-Stage Liver Disease, CHILD: Child–Turcotte–Pugh Score, TIPS+E: Transjugular Intrahepatic Portosystemic Shunt plus variceal Embolization, NA: Not Available, HR: Hazard Ratio, CI: Confidence Interval.

**Table 2:** Studies reporting results on the use of TIPS in bleeding from ectopic varices in patients with cirrhosis.

	Baseline							TIPS Procedure					Outcomes				
Study	Patients	TIPS Indication	Liver Disease Etiology	MELD and CHILD (%)	Type of Fundal Varix	PVT	Follow-Up	Stent	PPG (Pre–Post) Specify If Review Was Performed	Combination with Other Endovascular Procedure	Technical Success Rate	Stent Dysfunction Rate	TIPS-Related Complications (HE, Cardiac Failure, ecc)	Post-TIPS Rebleeding	Further Treatments for Rebleeding	Mortality	Relevant Details
Haskal, 1994, retrospective [96]	9	6 = active bleeding 2 = secondary prophylaxis 1 = pre-surgical	ALD 4 (44%) PBC 2 (22%) Cryptogenic 3 (34%)	MELD NA CHILD A/B/C 2/2/5 (22/22/56)	Jejuno-ileal = 6; Colonic = 3	1 patient with splenic and superius mesenteric vein thrombosis	Median 15 months (range, 9–21)	Bare Stent dilated to 10 mm	Pre = 26.8 ± 5.1 Post = 8.8 ± 2.9	Variceal embolization 2 (22%) Reason: NA 1 splenic/superior mesenteric vein percutaneous recanalization	100%	NA	HE 2 (22%) Cardiac failure = 2 (22%) Acute respiratory distress syndrome = 1 (11%)	1 (11%) at 24 h	Variceal embolization	55% at 6 months	One patient rebled despite post-TIPS PPG = 9 mmHg and previous partial variceal embolization → successfully treated with other feeders’ embolization Causes of death: - 2 = multiorgan failure/acute respiratory distress syndrome (at 5 days) - 2 = cardiac failure (at 2 and 6 months) - 1 = pneumonia
Shibata 1999, retrospective [98]	12	Active bleeding	ALD 5 (42%) HCV 2 (17%) HBV 1 (8%) PSC 2 (17%) Cryptogenic 1 (7%) Budd-Chiari 1 (7%)	MELD NA CHILD A/B/C 4/5/3 (33/42/25)	Anorectal = 7; Peristomal = 5	NA	Median 15 months (range 5–27)	Bare Stent dilated to 10 mm	Pre-TIPS = 17.4 ± 3.1 Post-TIPS = 5.8 ± 1.8	No	100%	33%	HE = 4 (33%) Immediate shunt thrombosis = 1 (8%) Hemoperitoneum = 1 (8%)	4 (33%)	TIPS revision	17% at 1 month	All rebleedings were in patients with stent dysfunction Causes of death: - 1 sepsis - 1 renal failure
Vangeli 2004, retrospective [95]	21	Active bleeding (13 = first episode; 8 = recurrent bleeding)	ALD 12 HCV 1 PSC 1 AIH 1 Cryptogenic 5 Post-transplant HBV 1	MELD 14.09 ± 9.58 CHILD 2/11/8 (10/52/38)	Rectal = 11; Stomal = 5; Duodenal = 1; Jejuno-ileal = 2 Colonic = 2	NA	Median 3 months (range 0–36)	Bare Stent dilated to 8, 10 or 12 mm to achieve PPG < 12 mmHg.	PPG measured with right atrial pressure. Pre-TIPS = 21 ± 5.7 Post-TIPS = 10.3 ± 4.6	Variceal embolization = 7 (33%) Reason: persistent variceal filling after PPG reduction < 12 mmHg (or by 25–50% of baseline)	90%	10%	HE = 2 (10%) Hemoperitoneum = 1 (5%)	7/19 (37%)	TIPS revision and variceal embolization	22% at 6 weeks; 26% and 3 months; 35% at 6 months	Five patients experienced early rebleeding despite reaching post-TIPS hemodynamic target → successfully treated with variceal embolization in 4 patients, 1 with surgical portocaval shunt Causes of death: sepsis and progressive liver failure
Vidal 2006, retrospective [99]	24	Active bleeding	ALD 13 (55%) HBV 1 (4%) PBC 2 (8%) PSC 1 (4%) AIH 1 (4%) Cryptogenic 5 (21%) Sarcoidosis 1 (4%)	MELD 13.3 (range 5.5–34.5). CHILD A/B/C 5/12/7 (21/50/29)	Stomal = 8; Ileocolic = 6; Duodenal = 5; Anorectal = 3; Umbilical = 1; Peritoneal = 1	NA	Median 592 days (range 28–2482)	Bare Stent dilated to 10 or 12 mm to achieve PPG < 12 mmHg.	Pre-TIPS = 19.7 ± 5.4 Post-TIPS = 6.4 ± 3.1	No	100%	51%	HE = 38% Hemoperitoneum = 8%; Haemobilia = 4% Bile leak = 4%	23% at 1 year and 31% at 2 years	TIPS revision and variceal embolization	20% at 1 year and 26% at 2 years.	Four patients experienced rebleeding despite reaching post-TIPS PPG < 12 mmHg → successfully treated with variceal embolization and 1 with surgical shunt Causes of death: 4 = liver failure 1 = bleeding from a sclerotherapy-induced rectal ulcer 2 = non-liver related
Kochar, 2008, retrospective [97]	28	Acute bleeding = 27 (25 first episode, 2 = recurrent bleeding) Secondary prophylaxis = 1	ALD 17 (60%) Viral 2 (7%) PSC 2 (7%) PBC 1 (4%) AIH 1 (4%) Cryptogenic 5 (18%)	MELD NA CHILD A/B/C 2/17/9 (7/61/32)	Rectal = 12 Stomal = 8 Duodenal = 4 Falciform ligament = 1 Caput medusa = 1 Intraperitoneal = 1 Mesenteric = 1	NA	Median 203 days (range 1–1869)	Covered stents (n = 8) 10 mm Bare stents (n = 19)	Pre-TIPS = 18.2 ± 6.4 Post-TIPS = 7.2 ± 3.5	Variceal embolization 5 = 18% Reason: significantly large collaterals with easily accessible feeding vessel	97%	15%	HE = 30% Heart failure = 7%	5/27 (21%) Cumulative risk of rebleeding at 1, 6, and 12 months was 13%, 21%, and 29%	TIPS revision, injection of thrombin, or variceal embolization	1-month (19%); 3-months(28%); 6-months (39%)	In 3 out of 9 patients with active bleeding at TIPS creation, bleeding could not be stopped, despite concomitant esophageal varices in two → no further treatment, early death (<5 days) Three patients experienced rebleeding despite reaching post-TIPS PPG < 12 mmHg → successfully treated with injection of thrombin in 2. Causes of death: - liver failure = 7 - HCC = 1 - uncontrolled bleeding = 3 - heart failure = 2 - sepsis = 1 - other = 2
Oey, 2018, retrospective [93]	53	Active bleeding	ALD 25 (47) Viral 2 (4) AIH/PBC/PSC 11 (21) Cryptogenic 7 (13) Other 8 (15)	MELD 11 CHILD A/B/C 34/15/3 (66/28/6)	Stomal = 23 * Duoneal = 12 Colon = 4 Rectum = 9 Jejunal = 1 Peritoneal = 3 Umbilical vein = 1 Peritoneal = 1	5 (9%)	Median 14 months (IQR 3.8–45.9)	Covered stents (n = 45) Bare stents (n = 8) Median dilatation 9 mm (8–10)	Pre-TIPS = 14 (IQR 10–20) Post-TIPS = 6 (IQR 4–7)	Variceal embolization 13 = 25% Reason: NA	100%	Covered stents 10/45 (22%) Bare stent 6/8 (75%)	HE = 30% Heart failure = 7%	12/53 (23%)	NA	41/53 (77%), the other 5 underwent liver transplant 1-month 11%, 1-year 41%, 5-year 75%.	* One patient presented with concomitant colostomy and urostomy bleeding Three patients experienced rebleeding despite reaching post-TIPS PPG < 12 mmHg Causes of death: - uncontrolled belleding = 3 - other liver related-causes = 9 - non-liver related = 12 -unknown = 17

Legend: ALD: Alcohol-associated Liver Disease, HCV: Hepatitis C Virus, HBV: Hepatitis B Virus, PBC: Primary Biliary Cholangitis, PSC: Primary Sclerosing Cholangitis, AIH: Autoimmune Hepatitis, HE: Hepatic Encephalopathy, TIPS: Transjugular Intrahepatic Portosystemic Shunt, PPG: Portal Pressure Gradient, HCC: Hepatocellular Carcinoma, IQR: Interquartile Range, MELD: Model for End-Stage Liver Disease, CHILD: Child–Turcotte–Pugh Score, NA: Not Available; * refers to important findings.

## Abbreviations

Acute variceal bleeding (AVB), portal hypertension (PH), transjugular intrahepatic portosystemic shunt (TIPS), gastric varices (GV), non-selective beta-blockers (NSBBs), balloon-occluded retrograde transvenous obliteration (BRTO), endoscopic band ligation (EBL), gastroesophageal varices type 1 (GOV1), self-expanding fully coated metal esophageal stent (SEMS), preemptive TIPS (pTIPS), Child–Turcotte–Pugh (CTP), gastro-renal shunt (GRS), retrograde transvenous obliteration (BRTO), antegrade transvenous obliteration (ATO), coil-assisted retrograde transvenous obliteration (CARTO), plug-assisted retrograde transvenous obliteration (PARTO).
